# Meniscal tears: comparison of the conventional spin-echo and fast spin-echo techniques through image processing

**DOI:** 10.1186/1475-925X-13-33

**Published:** 2014-03-27

**Authors:** Ibevan A Nogueira, Annie F Frère, Alessandro P Silva, Heverton C de Oliveira

**Affiliations:** 1 Núcleo de Pesquisas Tecnológicas, Universidade de Mogi das Cruzes, Mogi das Cruzes, São Paulo, Brasil; 2 Departamento de Diagnóstico por Imagem – EPM, Universidade Federal de São Paulo, São Paulo, Brasil

## Abstract

**Background:**

Conventional spin-echo (PD-CSE) and fast spin-echo (PD-FSE) techniques are frequently used to detect meniscal tears. However, the time delay for imaging with PD-CSE has resulted in its replacement with faster techniques, such as proton density fast spin-echo (PD-FSE), which has become a frequent tool at most diagnostic centres.

Qualitative analysis shows that the PD-CSE technique is more sensitive, but other authors have not found significant differences between the aforementioned techniques. Therefore, we performed a quantitative analysis in this study that aims to measure differences in the quality of the images obtained with both techniques.

**Methods:**

We compared the PD-CSE and PD-FSE techniques by quantitatively analysing the obtained proton density images: the area shown, as well as the brightness and lesion contrast of the obtained image.

A set of 100 images from 50 patients thought to contain meniscal tears of the knee were selected. These 100 images were obtained from all individuals using both the PD-CSE and PD-FSE techniques. The images were processed using software developed in Delphi. In addition to these quantifications, three physicians, who are specialists in radiology and capable of analysing magnetic resonance (MR) images of the musculoskeletal system, qualitatively analysed the diagnostic sensitivity of both techniques.

**Results:**

On average, samples obtained via the PD-CSE technique contained 22% more pixels in the lesion area. The contrast differed by 28%, and the brightness differed by 31%. The two techniques were correlated using Student’s t-test, which showed a statistically significant difference. The specialists detected meniscal tears in 30 of the images obtained via the PD-CSE technique, while only 72% of these cases were detected via the PD-FSE technique.

**Conclusions:**

The PD-CSE technique was shown to be superior to PD-FSE for all of the evaluated properties, making its selection preferable.

## Background

Magnetic resonance (MR) has manifested new horizons and perspectives in diagnosing musculoskeletal diseases, especially meniscal tears. MR images are peculiar during both the initial and later phases of meniscal tears because they show high resolution and excellent contrast between soft tissue structures. These images allow for rapid and precise diagnoses and thus decrease the need for the arthroscopic procedures that were historically used as the gold standard. Arthroscopic surgery is inherently difficult because it depends on much practice due to the narrow knee joint spaces and the complex anatomic structures [1]. However, inadequate MR techniques can mask these lesions [2]. Initially, the best diagnostic images were obtained using the proton density conventional spin-echo (PD-CSE) technique with fat saturation. This method was a pioneering technique for detecting meniscal tears and was considered to be a viable alternative to arthroscopy [3]. However, the time delay for imaging with PD-CSE resulted in its replacement with faster techniques, such as proton density fast spin-echo (PD-FSE), which has become a frequent tool at most diagnostic centres [4]. However, some researchers [5] find that the PD-FSE technique masks relevant details, as it loses over 10% of the image sensitivity, and suggest abandoning this technique. In contrast, other researchers [6] have not found significant differences between the two techniques and reported that PD-CSE provided only a small improvement in image quality. However, these studies were based on qualitative analyses. Therefore, our study aims to compare the diagnostic sensitivity of PD-CSE and PD-FSE techniques by quantifying the size, brightness and contrast of the imaged area using computerised processing of lesion images obtained with both techniques. Three physicians who have specialised in radiology with experience in magnetic resonance (MR) imaging of the musculoskeletal system qualitatively analysed the diagnostic sensitivity of both techniques.

## Methods

### Image bank

A set of 100 MR images was obtained from 50 male patients aged between 20 and 50 years who presented with suspected meniscal tears of the knee. These images were obtained using both the PD-CSE and PD-FSE techniques on the same individual during routine examinations after receiving patient consent (CAAE- 0042.0.237.000-08). The Lumen Clinic Diagnostics Centre (Clínica Lúmen Centro de Diagnósticos, Brazil) agreed to provide images already analysed by the responsible physician of 30 cases with meniscal tears and 20 without (Control Group).

The exams were performed using a Signa 1.5 T magnetic resonance machine (Signa LX: GE, Milwaukee, WI, USA) with a quadrature coil dedicated to knee studies and a 33 mT gradient coil. The conditions for the PD-FSE technique were a repetition time of 2260 ms and 2000 ms for the PD-CSE. For both techniques: echo time of 20 ms, a sectional thickness of 4.0 mm, an interval of 0.4 mm, a matrix frequency of 320, a phase of 256, a FOV (field of view) of 20 cm, 2 excitations (NEX), a bandwidth of 31.5 MHz. To PD-FSE was used a turbo factor of 4.

The images were collected in the DICOM format and transformed into BMP (bitmap).

### Qualitative analysis

Three physicians who specialised in the musculoskeletal system and are capable of analysing magnetic resonance exams qualitatively evaluated the images. The evaluators were shown the 100 images without any indication of the technique used. They were then asked to determine the images that showed meniscal tears, i.e., to provide a diagnosis. Their answers were recorded using a standard questionnaire that was manually filled out by the evaluators. To analyse the inter-observer reliability was used the contingency coefficient C.

### Quantitative analysis

The image processing software was developed using the Delphi v.7.0 language. A method for growing from a seed pixel was used to segment the region of interest, the meniscal tears. This method fills the region from an initial seed by analysing the direct neighbours [7]. This method performs well with small images without intersections between neighbouring regions. However, this method cannot easily automatically determine the growth boundary. In fact, finding a single value that works well for all lesions is difficult. We adopted an algorithm that adjusts the threshold value in an adaptive manner based on an estimate of the intensity of the external region [8].

Three meniscal lesion specialists validated these segmentations. These specialists compared the region marked by the computer to the lesions they identified. The developed software program also allowed a histogram of the region to be obtained. The contrast values were calculated from this histogram while considering the grey levels for pixels both within and outside the lesion. To define the contour, the border contrast was measured in 5 directions using line histograms for a total of 10 measurements, and the number of pixels in the segmented area was calculated.

The processing results were analysed using Student’s t-test to establish any significant differences between the analysed parameters for images obtained using the two techniques.

## Results

The evaluators analysed 100 images and detected lesions in 52 (physician 1), 51 (physician 2) and 52 (physician 3) images. After these evaluations, the images were categorised via the acquisition technique. The three evaluators found the same 30 images to certainly show meniscal tears for images obtained via the PD-CSE technique. The evaluators detected lesions in only 21 (physician 2) and 22 (physicians 1 and 3) of the 30 corresponding images obtained via PD-FSE. The average sensitivity was 72%. The contingency coefficient C showed which there was no significant difference in the inter-observer reliability (C = 0.0351, p = 0.94). In relation to previous images that had already been analysed (30 Positives and 20 Negatives). Meniscal tears were not detected in the 20 images of asymptomatic patients (False Positive). These images correspond to patients without lesions in the control group.

In general, the evaluators considered the PD-CSE images to provide greater clarity and contour definition. The quality criteria considered by the specialists were individually established as a function of their professional experience.

**Figure 1:**
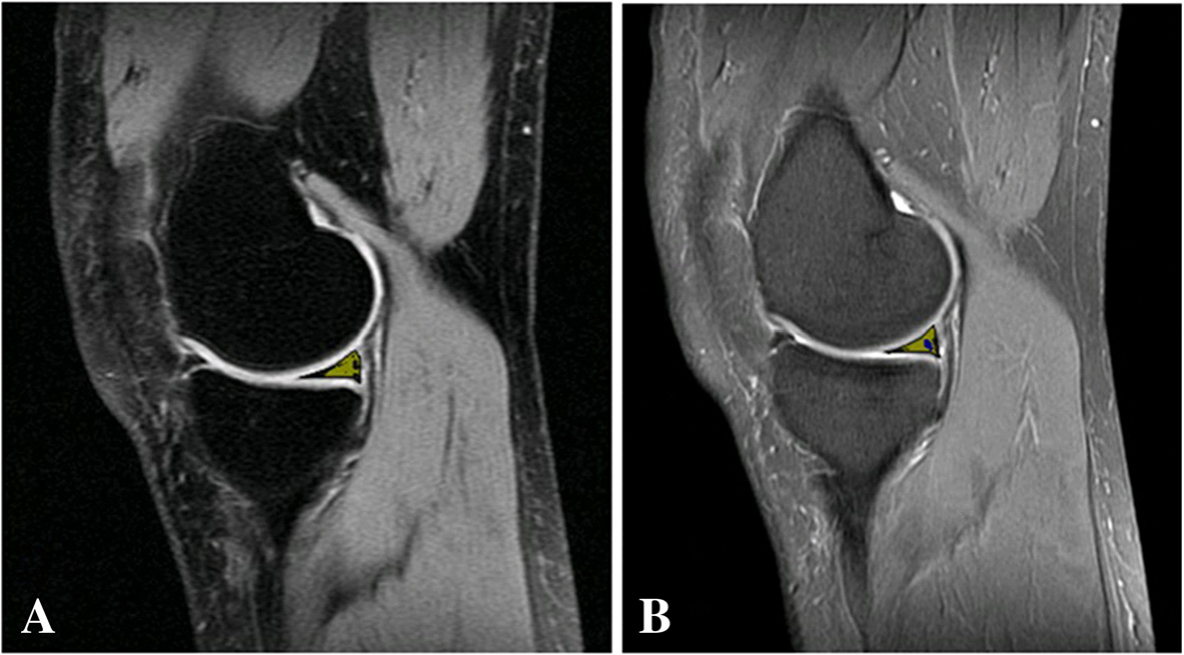
**Meniscal tears detection of volunteer 1.** Images from volunteer V1. The lesion area is marked in yellow. **A)** PD-CSE technique and **B)** PD-FSE technique.

**Figure 2:**
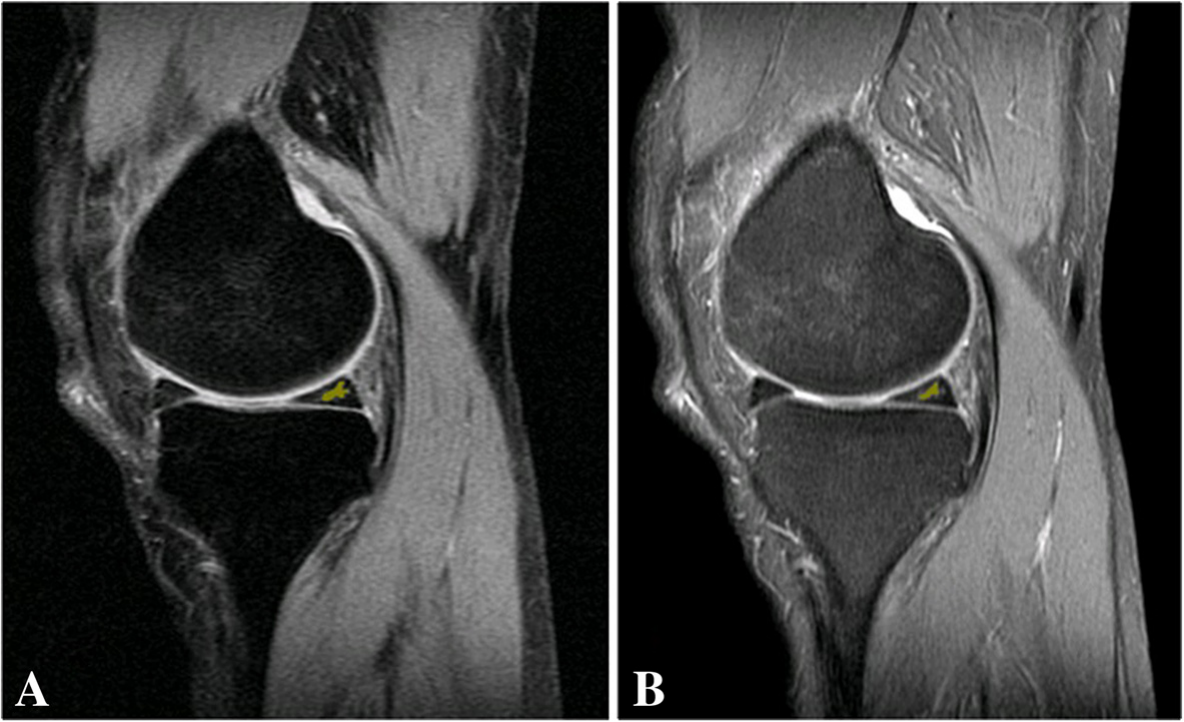
**Meniscal tears detection of volunteer 17.** Images from volunteer V17. The lesion area is marked in yellow. **A)** PD-CSE technique and **B)** PD-FSE technique.

**Figure 3:**
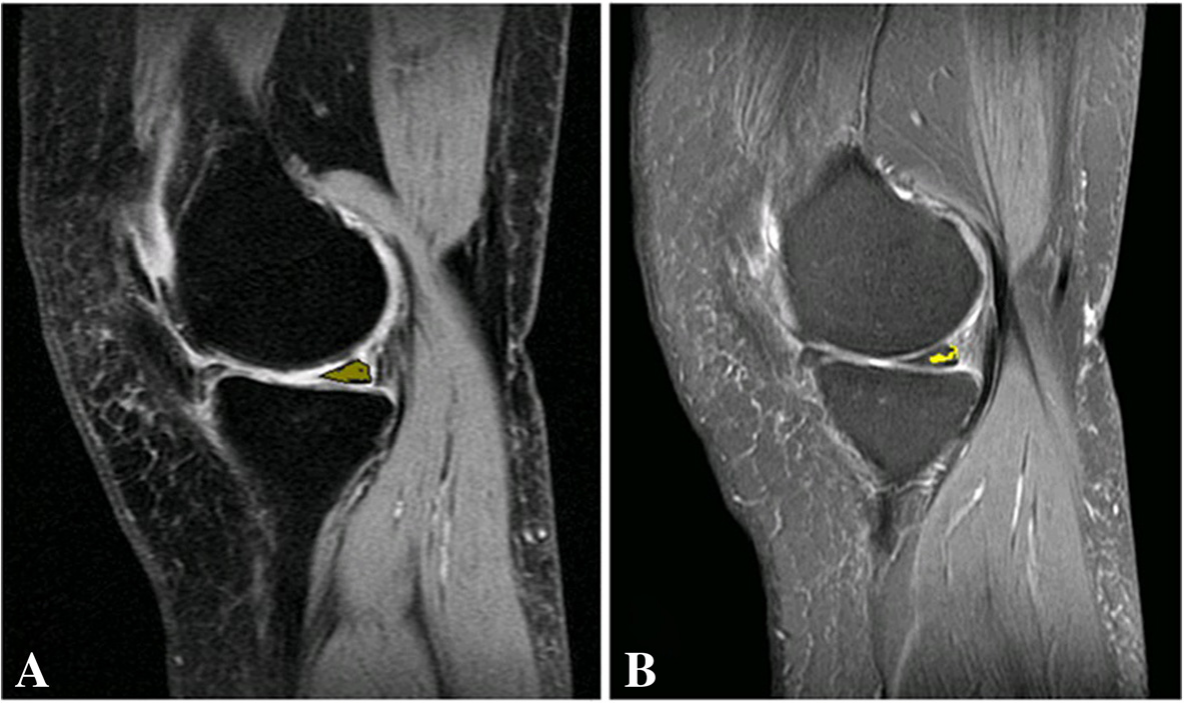
**Meniscal tears detection of volunteer 28.** Images from volunteer V28. The lesion area is marked in yellow. **A)** PD-CSE technique and **B)** PD-FSE technique.

**Figure 4:**
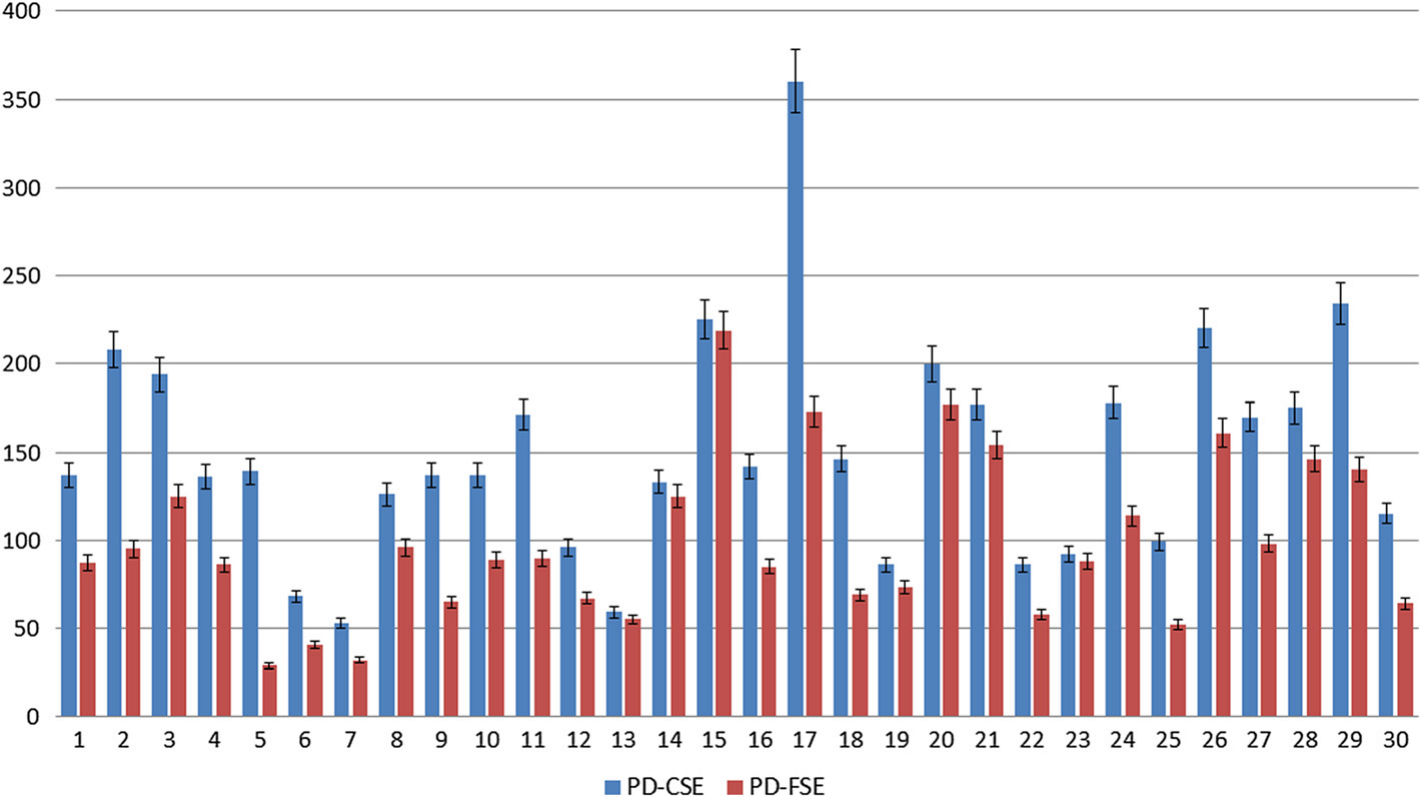
**Comparison of the pixel number between PD-CSE and PD-FSE.** Comparison of the pixel number for lesions in the 60 images obtained via PD-CSE and PD-FSE.

**Figure 5:**
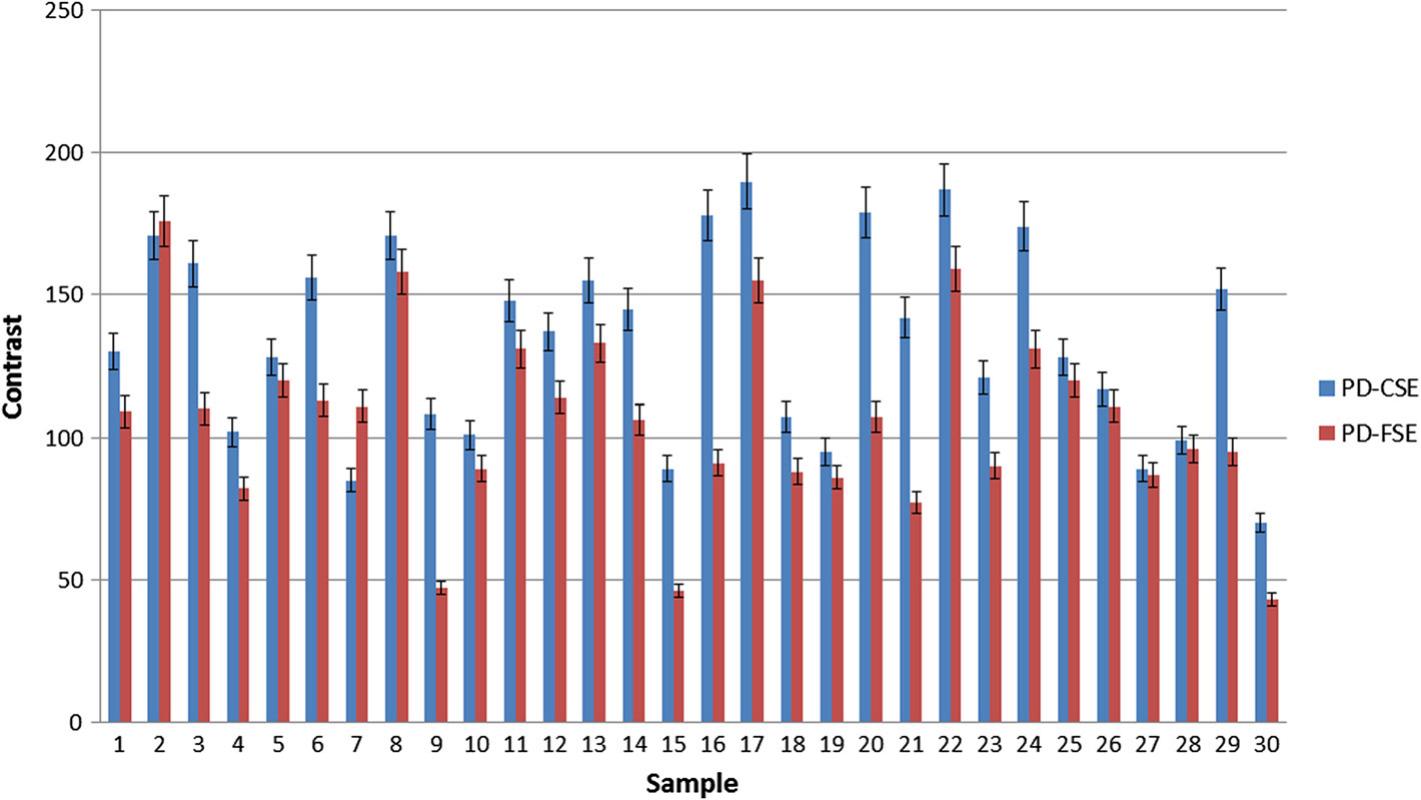
**Comparison of the contrast between PD-CSE and PD-FSE.** Comparison of the contrast between grey levels both within and outside the lesion for the 60 images obtained via PD-CSE and PD-FSE.

**Figure 6:**
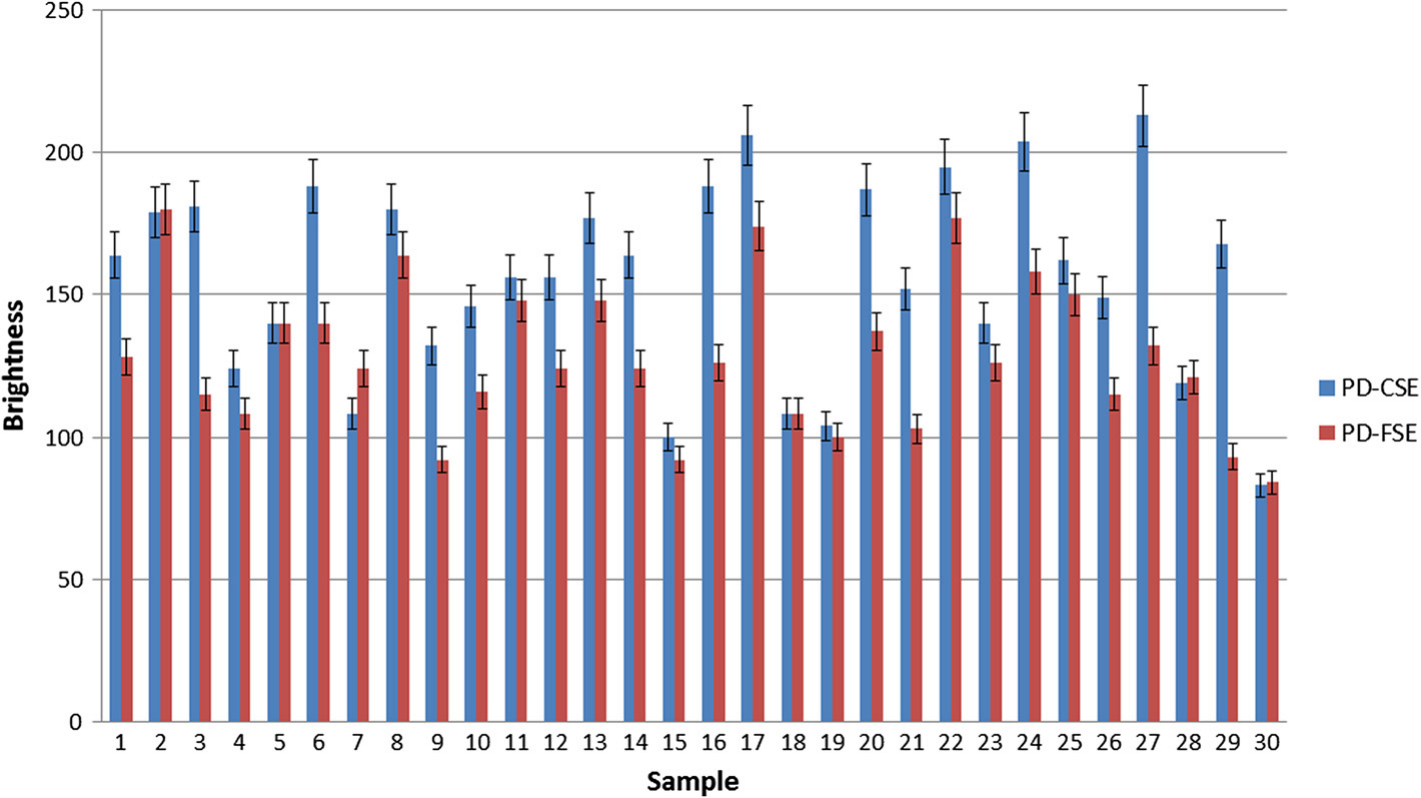
**Comparison of the brightness between PD-CSE and PD-FSE.** Comparison of the brightness of lesions for the 60 images obtained via PD-CSE and PD-FSE.

For the quantitative analysis, the 30 images identified as containing meniscal tears using the PD-CSE technique and the corresponding images obtained via PD-FSE were processed. To illustrate, Figures 1, 2 and 3 show the images obtained via PD-CSE and PD-FSE for volunteers V1, V17 and V28. In these images, the area marked during processing is highlighted. The 60 images were used to calculate the number of pixels, contrast and brightness in the marked region. The results for these images are shown in Figures 4, 5, and 6 as well as Table 1.

**Table 1:** Results of quantification

	**Contrast**		**Brightness**		**Number of pixel**	
**Sample**	**PD-CSE**	**PD-FSE**	**PD-CSE**	**PD-FSE**	**PD-CSE**	**PD-FSE**
1	130	109	164	128	137	87
2	171	176	179	180	208	95
3	161	110	181	115	194	125
4	102	82	124	108	136	86
5	128	120	140	140	139	29
6	156	113	188	140	68	41
7	85	111	108	124	53	32
8	171	158	180	164	126	96
9	108	47	132	92	137	65
10	101	89	146	116	137	89
11	148	131	156	148	171	90
12	137	114	156	124	96	67
13	155	133	177	148	59	55
14	145	106	164	124	133	125
15	89	46	100	92	225	219
16	178	91	188	126	142	85
17	190	155	206	174	360	173
18	107	88	108	108	146	69
19	95	86	104	100	86	73
20	179	107	187	137	200	177
21	142	77	152	103	177	154
22	187	159	195	177	86	58
23	121	90	140	126	92	88
24	174	131	204	158	178	114
25	128	120	162	150	99	52
26	117	111	149	115	220	161
27	89	87	213	132	170	98
28	99	96	119	121	175	146
29	152	95	168	93	234	140
30	70	43	83	84	115	64

Number of pixels, contrast and brightness for the lesion images obtained via PD-CSE and PD-FSE.

The paired Student’s t-test was applied to the image processing data generated using both techniques and resulted in t values of 7.1203 (p < 0.01) for the number of pixels (Figure 4), 6.1277 (p < 0.01) for the contrast (Figure 5) and 6.0553 (p < 0.01) for the brightness (Figure 6), i.e., the difference between the techniques was significant. A comparison of the pixel number shows a 22% greater area marked in the PD-CSE samples, a 28% difference in contrast, and a 31% difference in brightness.

## Discussion

In this study, evaluators of 100 representative knee images found a decreased accuracy in identifying meniscal tears when PD-FSE was used. These specialists found that PD-FSE resulted in a loss of clarity due to the blurring of the structures of interest as well as the reduced image size, brightness and contrast, which compromised the visualisation of small lesions. All of the experts found that the edges and lesion contours were better defined in PD-CSE images. However, these analyses are subjective. Currently, the applications of the analysis of variability are increasing in the medical field. A classification of these techniques has been widely discussed [9] and is applied in this study to better assess the sensitivity of each diagnostic method.

The quantification results from the image processing showed that the PD-CSE technique showed lesions with an average of 22% more pixels than those obtained via PD-FSE, which agreed with the opinion of the evaluating physicians. Moreover, the quantification showed that the first method yielded larger images. The 28% difference in lesion contrast confirms the opinion of the specialists that PD-CSE provides higher definition. The average 31% increase in brightness is consistent with the opinion of the physicians on clarity.

Previous studies in the literature [4-6,10] have compared the PD-CSE and PD-FSE techniques used to evaluate meniscal tears. These studies were based on arthroscopy procedures and visual observations by imaging specialists. Various researchers [4,6] did not find a significant difference between the two techniques and only referenced a small preference for the PD-CSE quality. Other authors [10] concluded that the PD-FSE technique is better because the technique is rapid and highly precise, specific, and sensitive for lesions on the medial poles and lateral portions of the meniscus. However, other authors [5] suggested that this technique might mask relevant details or generate doubts in the image presentation. These authors [5] found that meniscal tears are more visible in PD-CSE and recommend abandoning PD-FSE, having estimated a 10% loss of sensitivity; the quantification results from our study prove that PD-CSE images are more sensitive. A considerable increase in the grey scale was observed when comparing the frequencies, which indicates a greater sensitivity across the examined region. The results of this study bring into question the use of PD-FSE because it compromises or even prevents the detection of small lesions.

## Conclusions

This analysis of using PD-CSE and PD-FSE with fat saturation to evaluate meniscal tears found that the PD-CSE technique allowed detecting more lesions. The qualitative analysis results showed that PD-CSE was 28% more sensitive than PD-FSE. The PD-CSE technique is approximately 10% slower than PD-FSE. The significant difference in the image quality versus the relatively rapid image acquisition implies that this technique should not be used to detect micro meniscal tears of the knee.

## Competing interests

The authors declare that they have no competing interests.

## Authors’ contributions

IAN participated in the implementation of the software for the image analysis, concept and development of the study. AFF, APS and HCO also participated in the acquisition concept, analysis, and interpretation of the data. All authors revised and approved the current version of the manuscript.
